# The Effect of Xuefuzhuyu Oral Liquid on Aspirin Resistance and Its Association with rs5911, rs5787, and rs3842788 Gene Polymorphisms

**DOI:** 10.1155/2015/507349

**Published:** 2015-10-01

**Authors:** Mei Xue, Lin Yang, Na Kou, Yu Miao, Mingming Wang, Quanli Zhao, Junhua Ren, Shaoyan Zhang, Dazhuo Shi, Keji Chen

**Affiliations:** ^1^Cardiovascular Center, Xiyuan Hospital, China Academy of Chinese Medical Sciences, Beijing 100091, China; ^2^Physical Examination Center, Xiyuan Hospital, China Academy of Chinese Medical Sciences, Beijing 100091, China; ^3^Clinical Laboratory, The Affiliated Hospital of Qingdao University, Qingdao, Shandong 266033, China

## Abstract

Aspirin should be continued indefinitely in patients after interventional therapy, but 10% to 40% of patients experience recurrent vascular events despite adequate aspirin therapy, a condition known as aspirin resistance (AR). Xuefuzhuyu oral liquid, derived from the classic recipe Xuefuzhuyu decoction, has been well documented to inhibit platelet aggregation and to improve hemorheology. The aims of this study were to investigate the effects of Xuefuzhuyu oral liquid on AR in patients with chronic stable angina after percutaneous coronary intervention (PCI) and the possible genetic markers related to the drug response. 43 patients diagnosed as having aspirin resistance or semi-resistance were randomly divided into control and treatment groups after screening 207 stable CHD patients. Platelet aggregation rate was determined using turbidimetry. Three single nucleotide polymorphisms in COX-1 (rs5787, rs3842788) and GP IIb (rs5911) were genotyped in whole blood samples using ABI PRISM 7900 HT Fast Real-Time instrument and ABI PRISM 3730 DNA Sequencer. The results showed that Xuefuzhuyu oral liquid could effectively improve blood stasis syndrome and AR by inhibiting ADP-induced platelet aggregation and that patients with the rs5911 genetic variant exhibited better drug response upon treatment with Xuefuzhuyu oral liquid, which suggests Xuefuzhuyu oral liquid as a new possible drug for the prevention of AR.

## 1. Introduction

Platelet activation and aggregation have a pivotal role in the thrombotic complications that occur in patients undergoing percutaneous coronary intervention (PCI) [1]. Aspirin and clopidogrel (dual) antiplatelet therapy is recommended by the guidelines for the prevention of ischemic complications after PCI [2, 3]. However, bleeding events limit their clinical application. It is also recommended in the guidelines that if the risk of morbidity from bleeding outweighs the antiplatelet benefit of a recommended duration of P2Y12 inhibitor therapy after stent implantation, earlier discontinuation (<12 months) of P2Y12 inhibitor therapy is reasonable [4]. After PCI, aspirin administration should be continued indefinitely at a low dose. However, some patients still experience cardiovascular events despite its regular intake, a phenomenon which is known as aspirin resistance (AR) [5].

Gene polymorphisms can affect individual drug response. Detecting genetic variation may help to predict a patient's response to drugs and could be used as a tool to optimize therapy strategy, tailor dosage regimens, and improve clinical outcomes [6]. A number of studies have examined the association of AR with single nucleotide polymorphisms (SNPs) in the genes for COX-1 and for several receptors on the surface of platelets [7–9]. Maree reported COX-1 haplotypes (A-842G, C22T (R8W), G128A (Q41Q), C644A (G213G), and C714A (L237M)) were significantly associated with aspirin response determined by AA-induced platelet aggregation (*P* = 0.004; 4 d.f.) in patients (*n* = 144) with stable coronary heart disease (CHD) from Ireland [7]. Platelet glycoprotein (GP) IIb/IIIa receptors play an inevitable role in platelet aggregation [10]. Pamukcu reported that GP IIIa (PlA) polymorphism is related to aspirin resistance in Turkish patients with intracoronary stent restenosis, while our previous study has shown that there are only PlA1, A1 alleles of GP IIIa in 212 CHD patients and 39 healthy volunteers in the Chinese Han population [11, 12]. Therefore, it is important to find specific genetic markers for different ethnic groups.

Traditional Chinese medicines exhibiting good antiplatelet effects are the most commonly used drugs for patients after interventional therapy for activating blood circulation to remove blood stasis [13]. Xuefuzhuyu oral liquid, derived from the classic recipe Xuefuzhuyu decoction, can effectively inhibit platelet activation and reduce platelet aggregation and showed good effects in the clinical treatment of CHD [12]. But it still remains unknown whether Xuefuzhuyu oral liquid could relieve AR in patients after interventional therapy and if there are some specific gene polymorphisms related to the drug response. Therefore a control randomized study was designed to investigate the effects of Xuefuzhuyu oral liquid on AR in patients with chronic stable angina after PCI and the possible associated genetic markers for the drug response.

## 2. Materials and Methods

### 2.1. Patients

Patients were recruited from Xiyuan hospital, China Academy of Chinese Medical Sciences, from March 2012 to November 2014. The protocol was approved by the institutional Ethics Committee of China Academy of Chinese Medical Sciences, and all patients gave written informed consent. Trial is registered with Chinese Clinical Trial Register number ChiCTR-TRC-12002416.

### 2.2. Diagnostic Criteria

Chronic stable angina patients with coronary angiography showed stenosis ≥50% in at least one coronary artery or previous myocardial infarction [14]. Classification of CHD syndrome referred to the “Criterion of Syndrome Differentiation for CHD” by Cardiovascular Specialty Committee, China Association of Integrative Medicine [15]. Stasis syndrome differentiation and scores were made according to the “diagnostic criteria of blood stasis syndrome (BSS)” [16].

### 2.3. Inclusion and Exclusion Criteria

Inclusion criteria included (1) stable angina patients after postrevascularization or myocardial infarction, (2) 35 years ≤ age ≤ 75 years, (3) taking aspirin for more than 7 days, and (4) aspirin resistance. Aspirin resistance is defined when patients showed both (i) platelet aggregation rate ≥70% induced by diphosphate adenosine (ADP, 10 *μ*M) and (ii) platelet aggregation rate ≥20% induced by arachidonic acid (AA, 0.5 mg/mL). Aspirin semi-resistance is defined when either (i) or (ii) was observed. Exclusion criteria included (1) family or personal history of bleeding disorders, (2) platelet count <100 × 10^9^/L, or >450 × 10^9^/L, (3) hemoglobin <90 g/L, (4) taking other antiplatelet, anticoagulant drugs or nonsteroidal anti-inflammatory drugs, (5) taking other herbs besides Xuefuzhuyu oral liquid which are activating blood circulation to remove blood stasis within the latest two weeks, (6) history of trauma or surgery in the latest two weeks, (7) severe primary diseases like renal insufficiency, liver dysfunction, hematopoietic system diseases, mental disorder, or malignant tumor, and (8) female in pregnancy or lactation period.

### 2.4. Clinical Design and Treatment Procedure

The enrolled aspirin resistance or semi-resistance patients were randomly divided into control and treatment groups using a randomized block design. Conventional western medicine treatment and aspirin (100 mg) once daily were used in the control group, while Xuefuzhuyu oral liquid (10 mL, three times per day) was added in the treatment group for four consecutive weeks. The enrolled patients should not take antiplatelet, anticoagulant drugs, nonsteroidal anti-inflammatory drugs, and any other herbs activating blood circulation to remove blood stasis besides Xuefuzhuyu oral liquid during the research period. Biochemical indicators of liver and kidney function, platelet aggregation rate, and BSS scores were detected before and after treatment.

### 2.5. Xuefuzhuyu Oral Liquid Preparation

Xuefuzhuyu oral liquid (national medicine permit number Z10950063, batch number 1211010) was kindly provided by Jilin Aodong Yanbian Pharmaceutical Co., Ltd. (Jilin, China). It contains water extracts of semen* persicae*, safflower,* Angelica*, rhizoma* ligustici wallichii*, rehmanniae, rot of peony,* Achyranthes*,* Bupleurum*, fructus aurantii immaturus,* Platycodon grandiflorum*, and liquorice. The main active components used for quality control in Xuefuzhuyu oral liquid are paeoniflorin (≥1.4 mg/mL) and ferulic acid (≥0.15 mg/mL), which meet the requirement of China State Food and Drug Administration (the state drug standards number YBZ11722004) [17].

### 2.6. Platelet Aggregation Studies

Platelet aggregation rate was determined among different patients groups using turbidimetry (Platelet Aggregation Instrument, LBYNJ2, Beijing Lipusheng Co., China). The inducer of platelet aggregation was ADP (10 *μ*M, Chrono-log Co., Havertown, USA) and AA (0.5 mg/mL, Chrono-log Co., Havertown, USA).

### 2.7. DNA Preparation and Genotyping

Genomic DNA was isolated from whole blood using a Wizard Genomic DNA Purification Kit (Promega Co., USA) in accordance with the manufacturer's instructions as previously described [18]. Patients were genotyped for three single nucleotide polymorphisms in COX-1 (rs5787, rs3842788) and GP IIb (rs5911) (Table 1). Genotyping was performed using Taqman probe technique (rs5787 and rs5911) and gene sequencing technology (rs3842788) on an ABI PRISM 7900 HT Fast Real-Time instrument (Applied Biosystems, Foster City, CA) and an ABI PRISM 3730 DNA Sequencer (Applied Biosystems, Foster City, CA, USA), respectively, as has previously been described [12, 18].

### 2.8. Statistical Analysis

Continuous variables were expressed as means ± standard deviation (SD). One-way analysis of variance (ANOVA) was carried out for the comparison of means. Categorical data were described by frequency tables, percentage, or constituent ratio and analyzed by Chi-square test. All statistical analysis was performed with SPSS version 13.0, and *P* value of less than 0.05 was considered statistically significant.

## 3. Results

### 3.1. General Clinical Characteristics

43 patients diagnosed as having aspirin resistance or semi-resistance were randomly divided into control and treatment groups after screening 207 stable CHD patients, and there were no significant adverse reactions occurring before and after treatment. There was no statistical difference between the two groups in age, sex, and body mass index (*P* > 0.05). The risk factors (myocardial infarction, hypertension, dyslipidemia, and diabetes history) and statins medication history were comparable between the two groups (*P* > 0.05) (Table 2). All the patients enrolled carried only the G/G allele in both rs5787 and rs3842788 gene polymorphisms. The C haplotype of rs5911 was carried by 76.2% and 72.7% of patients in the two groups, respectively, without significant difference (*P* > 0.05).

### 3.2. Improvement of Aspirin Resistance before and after Treatment

90.5% of patients retained aspirin resistance or aspirin semi-resistance in the control group after conventional western medicine treatment, while only 18.2% retained resistance in the treatment group after combination therapy with Xuefuzhuyu oral liquid (*P* < 0.01) (Table 3).

### 3.3. Comparison of BSS Patients and BSS Scores between Groups

There were 12 patients (57.1%) and 18 patients (81.8%) with BSS, respectively, in the control and treatment groups with no statistical difference (*P* > 0.05) (Table 4). The BSS scores in the treatment group were reduced significantly after combination therapy with Xuefuzhuyu oral liquid compared to the control group, which indicated that Xuefuzhuyu oral liquid could reduce the degree of blood stasis in patients.

### 3.4. Correlation between Gene Polymorphism and the Effects of Xuefuzhuyu Oral Liquid on AR

ADP-induced platelet aggregation was significantly lower (*P* < 0.05, *P* < 0.01) after treatment in combination with Xuefuzhuyu oral liquid, no matter what kind of genotypes (A/A or A/C + C/C) the patients had, while AA-induced platelet aggregation provoked no significant change (Table 5), which indicated that Xuefuzhuyu oral liquid improves aspirin resistance by inhibiting ADP-induced platelet aggregation.

### 3.5. Comparison of BSS Scores in Patients with Different Genotypes before and after Treatment

After treatment in combination with Xuefuzhuyu oral liquid, BSS scores decreased significantly in patients with A/C or C/C genotype (Table 6), which illustrated that patients carrying the C allele were more responsive to the improvement of blood stasis symptoms and more sensitive to the treatment of Xuefuzhuyu oral liquid.

## 4. Discussion

An aspirin maintenance dose should be continued indefinitely in patients after interventional therapy, and it was reported that aspirin could reduce serious vascular events by 25% in patients with high risk conditions [19]. However, its effectiveness is limited because 10% to 40% of patients with arterial thrombosis who are treated with aspirin have recurrent vascular events during long-term follow-up [20]. Eikelboom reported that AR patients, defined as failure of suppression of thromboxane generation, had a 2-times-higher risk of myocardial infarction and a 3.5-times-higher risk of cardiovascular death than those with low expression of thromboxane [21]. It has been suggested that higher doses of aspirin or dual antiplatelet therapy may be required in AR patients to achieve the optimal antithrombotic effect [22]. However, bleeding and upper gastrointestinal damage have been serious complications of this therapeutic strategy with high morbidity and mortality [23, 24]. Therefore, it is urgent to find novel effective and safe antiplatelet agents, which provides a great opportunity for traditional Chinese medicine with multitarget effects.

Xuefuzhuyu oral liquid, a Chinese herbal patent medicine (containing water extracts of semen* persicae*, safflower,* Angelica*, rhizoma* ligustici wallichii*, rehmanniae, rot of peony,* Achyranthes*,* Bupleurum*, fructus aurantii immaturus,* Platycodon grandiflorum*, and liquorice) approved by the China State Food and Drug Administration (national medicine permit number Z10950063), has been used in the treatment of ischemic cardiovascular diseases in mainland China for more than 20 years [17]. Xuefuzhuyu oral liquid, derived from the classic recipe Xuefuzhuyu decoction, has been well documented to inhibit platelet aggregation and to improve hemorheology [13]. In the present study, 43 enrolled patients with chronic stable angina after PCI exhibiting aspirin resistance or semi-resistance were randomly divided into control and treatment groups using a randomized block design. Only 18.2% of patients retained AR or ASR in the treatment group after combination therapy with Xuefuzhuyu oral liquid, while 90.5% retained resistance in the control group, which illustrated that Xuefuzhuyu oral liquid could effectively improve AR in patients with chronic stable angina after PCI. The BSS scores in the treatment group were reduced significantly after combination therapy with Xuefuzhuyu oral liquid compared to the control group, which indicated that Xuefuzhuyu oral liquid could reduce the degree of blood stasis in patients. Aspirin exerts its major antithrombotic effect by irreversibly acetylating platelet cyclooxygenase-1 (COX-1). One of the possible explanations for AR is that platelets can be activated by pathways that are not blocked by aspirin [21]. ADP-induced platelet aggregation was significantly lower after treatment in combination with Xuefuzhuyu oral liquid, while AA-induced platelet aggregation provoked no significant change, which demonstrated that Xuefuzhuyu oral liquid improves AR by inhibiting ADP-induced platelet aggregation. But the possible mechanism of Xuefuzhuyu oral liquid on improving AR remained unknown. Because the main active components in Xuefuzhuyu oral liquid are considered to be paeoniflorin and ferulic acid, which are reported to have good antiplatelet effects [17], the further study on the mechanisms may be focused on the ADP-pathway of these ingredients.

Many researches are currently focusing on identifying variants of genes that affect drug response. Because aspirin exhibited antiplatelet aggregation effects by irreversible inhibition of COX-1, polymorphisms of the COX-1 gene are in the focus of many researches, but the roles of COX-1 SNPs in the mechanism of AR have not been fully elucidated. rs3842788 has been shown to be significantly associated with aspirin response determined by AA-induced platelet aggregation and serum TXB2 generation in Irish patients with cardiovascular disease, while Xu et al. reported that the mutation of rs3842788 (4.44% mutant) was not related to AR in patients accepting aspirin treatment in China [7, 8]. An in vitro study proved rs5787 variants exert the largest functional effects on decreasing the antiplatelet effectiveness of aspirin among four SNPs, with evidence for impaired interactions with COX substrate and inhibitors [25]. In our present study, no variants of rs5787 and rs3842788 were detected among 207 stable CHD patients enrolled, and because the mutations of rs5787 and rs3842788 were very uncommon, they were not suitable as representative gene polymorphisms for AR in the Chinese population. The GP IIb/IIIa receptor is critical in the process of thrombus formation since it serves as the final common pathway for platelet aggregation [26]. Several polymorphisms of the GP IIb/IIIa receptor have been identified in the general population. As compared to rs5911 C/C homozygotes, individuals with the rs5911 A/C genotype showed significantly increased inhibition of platelet aggregation in healthy Chinese male volunteers [27]. In the present study, BSS scores decreased significantly in patients with A/C or C/C genotype after treatment with Xuefuzhuyu oral liquid, which showed that patients carrying the C allele were more responsive to the improvement of blood stasis symptoms and more sensitive to the treatment of Xuefuzhuyu oral liquid.

Therefore, Xuefuzhuyu oral liquid therapy in addition to aspirin administration for the treatment of chronic stable angina patients after PCI leads to greater protection from AR, and the patients with the rs5911 variants of GP IIb exhibited better drug response upon treatment with Xuefuzhuyu oral liquid. More rigorous randomized controlled trials are necessary to provide clinicians with evidence regarding the use of Xuefuzhuyu oral liquid in the treatment of AR.

## Supplementary Material

Quality Inspection Report of Xuefuzhuyu Oral Liquid.
The main active components used for quality control in Xuefuzhuyu oral liquid (national medicine permit number Z10950063, batch number 1211010) are paeoniflorin (=1.4 mg/mL) and ferulic acid (=0.15 mg/mL), which meet the requirement of China State Food and Drug Administration (the state drug standards number YBZ11722004).

## Figures and Tables

**Table 1:** COX-1 and GP IIb single nucleotide polymorphisms.

	Region	Contig position	mRNA position	dbSNP rs cluster id number	RefSNP allele	Protein residue	Codon position	Amino acid position
COX-1	Exon_4	32462028	458	rs5787	G	Arg [R]	2	108
					A	Gln [Q]	2	108
	Exon_3	32461411	258	rs3842788	A	Gln [Q]	3	41
					G	Gln [Q]	3	41

GP IIb	Exon_26	1106870	2653	rs5911	A	Ile [I]	2	874
					C	Ser [S]	2	874

Notes: dbSNP: single nucleotide polymorphism database; RefSNP: reference single nucleotide polymorphism.

**Table 2:** Baseline characteristics of study participants.

	Control group (*n* = 21)	Treatment group (*n* = 22)
Age, y	62.8 ± 6.3	67.0 ± 8.1
Male sex, *n* (%)	6 (28.6)	10 (45.5)
Body mass index, kg/m^2^	24.7 ± 2.9	26.9 ± 6.1
Statins, *n* (%)	15 (71.4)	15 (68.2)
Myocardial infarction history, *n* (%)	3 (14.3)	5 (22.7)
Hypertension, *n* (%)	14 (66.7)	18 (81.8)
Dyslipidemia, *n* (%)	16 (76.2)	16 (72.2)
Diabetes, *n* (%)	10 (47.6)	11 (50)
rs5787 GG, *n* (%)	21 (100)	22 (100)
rs5911 AA/(AC + CC)	5/16	6/16
rs3842788 GG, *n* (%)	21 (100)	22 (100)

**Table 3:** Improvement of aspirin resistance before and after treatment.

	Aspirin resistance/aspirin semi-resistance	
	Before treatment *n* (%)	After treatment *n* (%)
Control group	21 (100)	19 (90.5)
Treatment group	22 (100)	4 (18.2)^*∗∗*∧∧^

Notes: ^*∗∗*^ *P* < 0.01, compared with the control group; ^∧∧^ *P* < 0.01, compared with before treatment.

**Table 4:** Comparison of BSS patients and BSS scores between groups.

	BSS patients *n* (%)	Non-BSS patients *n* (%)	BSS scores	
			Before treatment	After treatment
Control group	12 (57.1)	9 (42.9)	20.25 ± 5.59	19.92 ± 4.81
Treatment group	18 (81.8)	4 (18.2)	21.11 ± 3.38	13.5 ± 3.36^*∗*∧^

Notes: ^*∗*^ *P* < 0.05, compared with the control group; ^∧^ *P* < 0.05, compared with before treatment.

**Table 5:** Effects of Xuefuzhuyu oral liquid on patients' platelet aggregation with different genotyping of rs5911.

rs5911 genotyping	Group	*n*	ADP-induced platelet aggregation rate	AA-induced platelet aggregation rate
A/A	Before treatment	6	77.06 ± 6.48	12.21 ± 7.17
	After treatment	6	63.65 ± 4.27^*∗∗*^	11.48 ± 4.73

A/C + C/C	Before treatment	16	72.09 ± 14.20	16.38 ± 7.18
	After treatment	16	60.88 ± 13.37^*∗*^	14.03 ± 3.32

Notes: ^*∗*^ *P* < 0.05 and ^*∗∗*^ *P* < 0.01, compared with before treatment.

**Table 6:** Comparison of BSS scores in patients with different genotypes before and after treatment.

rs5911 genotyping	Group	*n*	BSS scores
A/A	Before treatment	8	19.88 ± 3.23
	After treatment	8	15.75 ± 3.65

A/C + C/C	Before treatment	22	21.09 ± 4.69
	After treatment	22	16.18 ± 5.56^*∗∗*^

Notes: ^*∗∗*^ *P* < 0.01, compared with before treatment.
