# Supplementary figures and images for: The Effect of Xuefuzhuyu Oral Liquid on Aspirin Resistance and Its Association with rs5911, rs5787, and rs3842788 Gene Polymorphisms
Source: Evid Based Complement Alternat Med. 2015 Oct 1;2015:507349. doi: 10.1155/2015/507349 (PMC4606155; doi:10.1155/2015/507349)

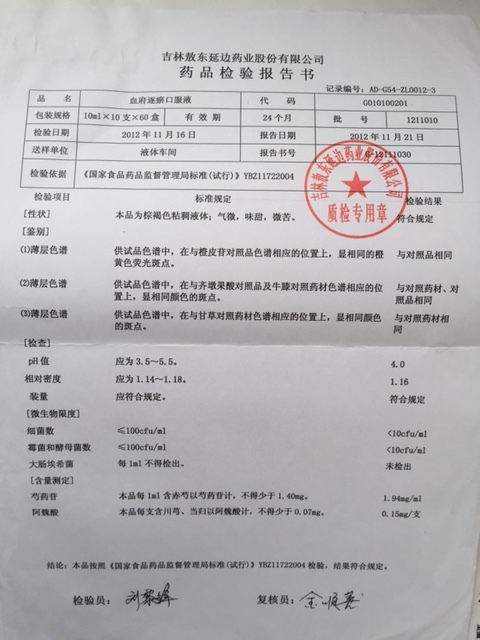

Supplement: Supplementary file 1 — Quality Inspection Report of Xuefuzhuyu Oral Liquid. The main active components used for quality control in Xuefuzhuyu oral liquid (national medicine permit number Z10950063, batch number 1211010) are paeoniflorin (=1.4 mg/mL) and ferulic acid (=0.15 mg/mL), which meet the requirement of China State Food and Drug Administration (the state drug standards number YBZ11722004). [file 507349.f1.jpg]
